# Functional hair follicle regeneration: an updated review

**DOI:** 10.1038/s41392-020-00441-y

**Published:** 2021-02-17

**Authors:** Shuaifei Ji, Ziying Zhu, Xiaoyan Sun, Xiaobing Fu

**Affiliations:** Research Center for Tissue Repair and Regeneration affiliated to the Medical Innovation Research Department and 4th Medical Center, PLA General Hospital and PLA Medical College; PLA Key Laboratory of Tissue Repair and Regenerative Medicine and Beijing Key Research Laboratory of Skin Injury, Repair and Regeneration; Research Unit of Trauma Care, Tissue Repair and Regeneration, Chinese Academy of Medical Sciences, 2019RU051, Beijing, 100048 People’s Republic of China

**Keywords:** Regeneration, Regenerative medicine

## Abstract

The hair follicle (HF) is a highly conserved sensory organ associated with the immune response against pathogens, thermoregulation, sebum production, angiogenesis, neurogenesis and wound healing. Although recent advances in lineage-tracing techniques and the ability to profile gene expression in small populations of cells have increased the understanding of how stem cells operate during hair growth and regeneration, the construction of functional follicles with cycling activity is still a great challenge for the hair research field and for translational and clinical applications. Given that hair formation and cycling rely on tightly coordinated epithelial–mesenchymal interactions, we thus review potential cell sources with HF-inducive capacities and summarize current bioengineering strategies for HF regeneration with functional restoration.

## Background

**Fig. 1:**
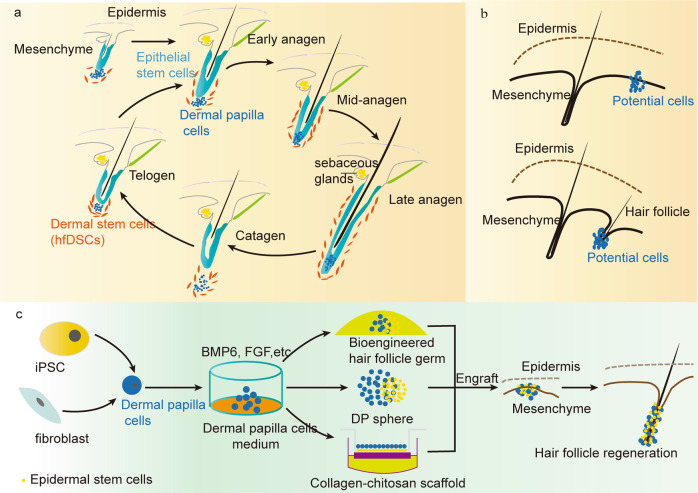
The process of hair cycling and DPCs in HF regeneration. **a** Mature and actively growing HFs anchored in the subcutis periodically regenerate by spontaneously undergoing anagen (repetitive cycles of growth), catagen (apoptosis-driven regression) and telogen (relative quiescence), which is termed hair cycling and is a typical characteristic of functional HFs. **b** Skeleton diagram of potential cells that contribute to regenerating HFs. **c** iPSCs share similar characteristics with embryonic stem cells in terms of morphology, self-renewal and differentiation capacity, and they can be induced into other potential cells in regenerative medicine. The transformation of fibroblasts into DPCs via lineage reprogramming. Optimization of the in vitro system to preserve the HF-inducive potential and the transplantation of cell-based biomaterials or HF organoids in vivo to regenerate HFs

**Fig. 2:**
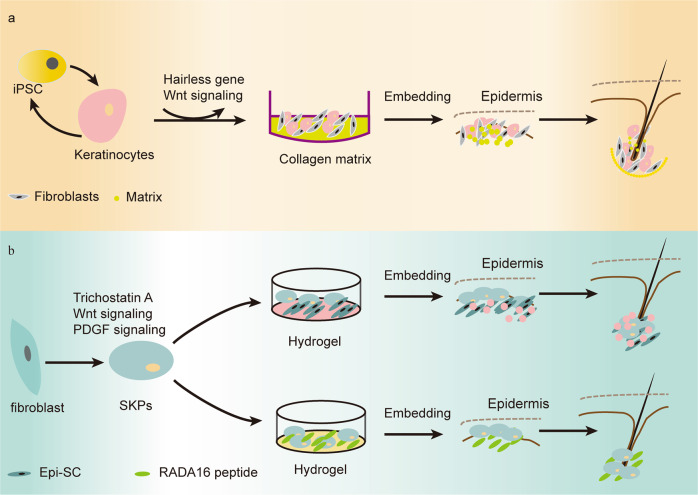
Keratinocytes and SKPs in HF regeneration. **a** iPSCs were reprogrammed into keratinocytes, and biomaterials containing a mixture of fibroblasts and keratinocytes were embedded for de novo HF. **b** Fibroblasts were chemically induced into SKPs. A mixture of SKPs and epidermal stem cells or bioactive peptides was embedded in the hydrogel to regenerate HFs three-dimensionally

**Fig. 3:**
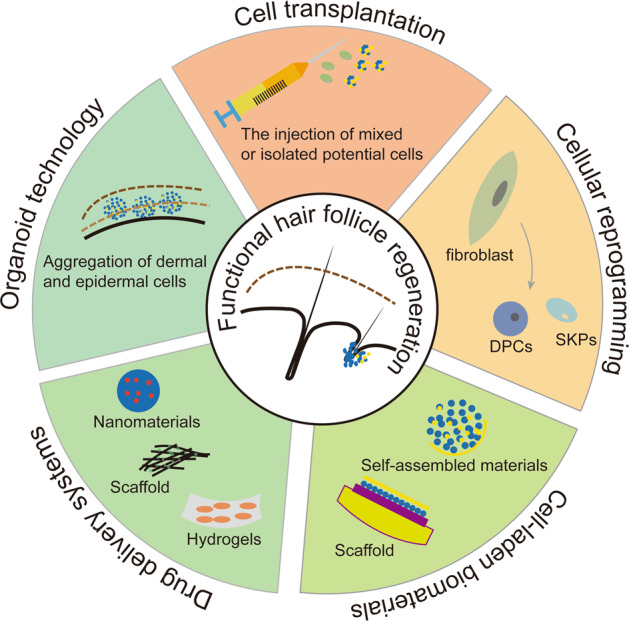
Strategies to achieve functional HF regeneration

Hair follicles (HFs) are a major skin appendage originating from the ectoderm. As a stem cell repository and a hair shaft factory, the HF contributes to remodelling its cutaneous microenvironment, including skin innervation and vasculature.^1^ The HF participates in multiple functions, mainly physical protection, thermal insulation, camouflage, sebaceous dispersion, sensory perception and social interactions. In addition, hair in human society greatly affects the quality of life, attractiveness and self-esteem. However, destructive inflammation with various aetiologies and the subsequent replacement of fibres can involve the permanent loss of HFs, which impairs inherent skin function and, especially, psychological well-being. Thus, HF regeneration is in ever-increasing demand and has promising market prospects. HF morphogenesis and regeneration were shown to be dependent on the intensive cooperation of epithelial (epidermal stem cell [Epi-SCs]) and hair-inducive mesenchymal (dermal papilla [DP]) components, also called epithelial–mesenchymal interaction (EMI). EMI is a prerequisite for functional HF formation, regeneration and cycling, mainly through paracrine mechanisms,^2^ and has become the theoretical basis of tissue engineering for HF regeneration. Current strategies to regenerate HF in vivo are aimed at simulating EMI, mostly adopting the principle of combining epithelial (Epi-SC and keratinocyte) and mesenchymal (DP cell [DPC] and skin-derived precursors [SKPs]) components. HFs are also a dynamic mini-organ, and their most notable feature is hair cycling (Fig. 1a), from periods of organ regeneration and rapid growth (anagen) to apoptosis-driven regression (catagen); then, the HF moves back into anagen via an interspersed period of relative quiescence (telogen).^1^ The interaction between HF stem cells (HFSCs) and DPCs plays a significant role in the regulation of hair cycling.^3,4^ The activation, stability and sustainability of hair cycling are considered to be a key factor in achieving the longevity of HF function, but HF loss is often accompanied by the termination and elimination of hair cycling. Thus, the achievement of hair cycling regeneration is important for functional HF regeneration. Although hair transplantation has been widely applied, transplanted hair is not maintained in the long term. Moreover, clinical drugs still fail to meet the patents’ needs and even have drastic side effects.^5^ Therefore, there is a need to explore alternative therapeutic solutions capable of generating functional HFs. Current techniques could make it possible to obtain potential cells in vitro (Fig. 1b), such as DPCs (Fig. 1c), SKPs, keratinocytes and other stem cells (Fig. 2), providing us with a series of cell sources. In addition, the optimization of the culture system also contributes to preserving the HF-inducive ability of potential cells. Based on these findings, we are able to regenerate functional HFs by the transplantation of potential cell mixtures, HF organoid construction in vitro, reprogramming induction and the establishment of a drug delivery system (Fig. 3). Here, we will review the potential cell sources and tissue engineering techniques that contribute to HF regeneration. In addition, the limitations and future of functional HF regeneration are summarized.

## Potential cell sources and mechanism for HF regeneration

### DP cells

DPCs, a kind of differentiated dermal cell at the base of HFs,^6^ originate from blimp1+ fibroblasts (dermal stem cells [DSCs]) during embryonic development.^7,8^ DPCs have the ability to stimulate epithelial HFSCs and are considered to be a master regulator of HF cycling. Past studies reported that DPCs isolated from rat and guinea pig vibrissae, as well as humans, could also induce HF formation when implanted into recipient non-hairy skin,^9,10^ which indicates that DPCs could reprogramme non-hairy epidermis to a follicular fate. Subsequently, DPCs, either fresh or after tissue culture expansion, could also reproduce new HFs if placed in proximity to the epithelium.^11,12^ Based on their strong HF-inducive ability, many attempts to coculture DPCs with other cell types to regenerate HFs have been studied, such as the two-dimensional juxtaposition of other epithelia,^13^ cultured epithelial cells,^14,15^ keratinocytes,^16,17^ corneal epithelium^18^ and amnion epithelium.^19^ DPCs can secrete various factors to initiate HF formation by activating skin epithelial stem cells (Epi-SCs), so the mixture of DPCs and Epi-SCs also promotes functional HF regeneration in vivo.^20^

**Fig. 4:**
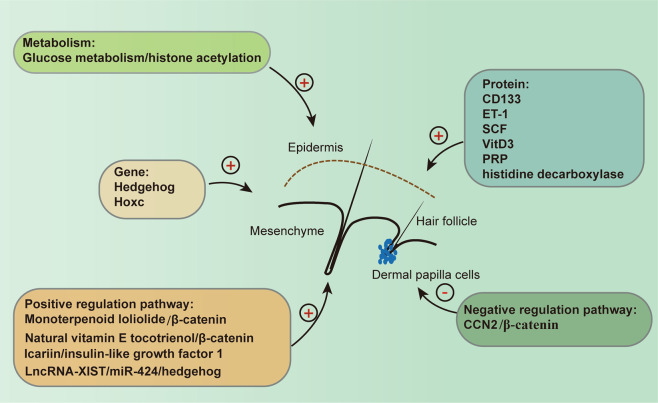
The regulatory factors of DPC HF-inducive capacity. Glucose metabolism and LncRNA-XIST/miR-424 axis in the regulation of HF-inducive potential of DP spheres

DPCs with specific marker molecules possess HF-inducive capacity, including CD133^+^ DPCs and Versican^+^ DPCs. CD133^+^ DPCs have been shown to be a specific subpopulation of cells in DPCs, and they can produce Wnt ligands and mediate signalling crosstalk between the mesenchyme and the epithelial compartment, further promoting adult HF growth and regeneration.^21^ In addition, Versican^+^ DPCs exhibit the typical characteristics of aggregation growth,^22^ on which HF formation is highly dependent.^23^ In addition, many functional molecules are involved in the positive regulation of DPC HF-inducive capacity (Fig. 4), such as *endothelin-1 and stem cell growth factor*,^24^ *insulin-like growth factor-1* *(IGF-1)*^25^ and *histidine decarboxylase*,^26^ but *matricellular protein connective tissue growth factor (CCN2)* negatively regulates HF regeneration, physiologically curbing HF formation by the destabilization of *β-catenin*.^27^ In wound healing, *hedgehog* gene activation could shift the dermal fibroblast fate towards DPCs and result in extensive HF neogenesis.^28^ *Hoxc* genes are able to reprogramme DPCs, and a single *Hoxc* gene is sufficient to activate dormant DP niches and promote regional HF regeneration through canonical Wnt signalling.^29^ Monoterpenoid loliolide regulates the HF inductivity of human DPCs by activating the AKT/β-catenin signalling pathway.^30^ Although DPCs possess the potential to regenerate HFs, freshly isolated human DPCs are not efficient in regenerating new HFs when they are directly transplanted.^31^ To restore their intrinsic properties, three-dimensional (3D) spheres offer a more physiologically relevant system where cell–cell communication as well as microenvironments have been studied. It has been reported that sphere formation increases the ability of cultured human DPCs to induce HF from mouse epidermal cells,^32^ in which glucose metabolism^33^ and epigenetics^34^ may be important regulators. The natural vitamin E form tocotrienol acts upstream of DP formation to induce HF anagen, dependent on the loss of E-cadherin and activation of β-catenin.^35^ Pretreatment with 1α,25-dihydroxyvitamin D3 (VitD3) could significantly improve DPC functionality and hair folliculogenesis, which is mediated by the activation of Wnt10b, alkaline phosphatase (ALP) and transforming growth factor β2 (TGF-β2).^36^ Consistent with VitD3, platelet-rich plasma has been shown to function in HF regeneration by enhancing DPC proliferation,^37^ which may result from the downregulation of *MYC, CCAAT/enhancer-binding protein beta* and E2F transcription factor-1 gene.^38^ Icariin promotes mouse HF growth by increasing IGF-1 secreted by DPCs.^39^ Utilization keratinocyte-conditioned medium,^40^ coculture with keratinocytes^40,41^ or the addition of BMP6^42^ and basic fibroblast growth factor (FGF)^43^ to DPC expansion cultures could preserve their HF-inducive capacity. JAK inhibitor regulates the activation of key HF populations, such as the hair germ, and improves the inductive potential of DPCs by controlling a molecular signature enriched in intact, fully inducive DPs.^44^

### Skin-derived precursors

DSCs are stem cells located in the dermis of the skin. Based on distinct phenotypic properties and different cultural environments, DSCs can be divided into dermal fibroblasts and SKPs.^7,45^ For instance, DPCs are differentiated dermal cells originating from blimp1^+^ dermal fibroblasts,^7^ while SKPs are derived from Sox2^+^ follicle-associated dermal precursors.^46^ SKPs are defined as DSCs that reside in the adult HF mesenchyme and can be isolated and expanded in vitro as self-renewing colonies. SKPs have the capacity to differentiate in vitro and in vivo into multiple lineages of different progeny.^47^ Of note is that SKPs could regenerate the dermal sheath and repopulate DPCs in each growth cycle^48^ to serially reconstitute HF when subcutaneously engrafted.^46^ There are also various molecules affecting the inductive potential of SKPs. Trichostatin A, a potent and specific inhibitor of a histone deacetylase, could restore the HF-inducive capacity of SKPs by markedly alleviating culture expansion-induced SKP senescence, increasing the expression and activity of ALP and elevating the acetylation level of histone H3.^49^ Platelet-derived growth factor (PDGF) could enhance SKP proliferation, increase SKP progeny and improve their HF-inducive capacity, but PDGF deficiency results in a progressive depletion of the stem cell pool and their progeny.^50^ Many tentative methods to isolate and cultivate SKPs have also been explored,^51,52^ particularly in computer-controlled stirred suspension bioreactors, which lead to a greater expansion of viable SKPs.^53^ This technique allows a large number of SKPs that share a similar expression profile to that in static culture (fivefold greater than in static culture), and both static and bioreactor condition-derived SKPs are able to induce de novo HFs and repopulate their cellular niches.^53^

### Epidermis-derived cells

HFSCs are heterogeneous Epi-SCs compartmentalized along the longitudinal axis of HFs. They are divided into quiescent and primed HFSCs. For instance, relatively quiescent HFSCs in the follicular bulge region can serve as a reservoir for transient amplifying cells that are able to produce various cell types during HF regeneration.^54^ Primed HFSCs have faster activation dynamics in the secondary hair germ of telogen HF.^55^ The activation of primed HFSCs could trigger the subsequent activation of quiescent HFSCs, and the coordination of these two populations could fuel HF regeneration from telogen to anagen.^56,57^ HFSCs express a panel of transcription factors, including Sox9, Tcf3, Lhx2 and Nfatc1.^57–59^ HFSCs derived from a single rat vibrissa via organ culture could reconstitute HFs in vivo,^60^ and even aged HFSCs still have the potential to regenerate HFs.^61^ HFSCs could also differentiate into epidermal and sebaceous gland lineages, participating in the process of skin wound healing, and thus were considered ideal candidates for cutaneous repair and regeneration. Recent studies have reported that basal keratinocytes also have the capacity to facilitate HF regeneration under induction. Transgenic expression of the hairless gene (*HR*) in progenitor keratinocytes could rescue HF regeneration in Hr(−/−) mice, in which the new HFs resemble wild-type follicles and express markers of early anagen.^62^ This process may be linked to the regulation of the precise timing of WNT signalling to HR.^62,63^ Tuberous sclerosis complex 2 (TSC2)-null fibroblast-like cells grown from human TSC skin hamartomas could also induce neonatal foreskin keratinocytes to form HFs, complete with sebaceous glands, hair shafts, inner and outer root sheath (ORS) cells and the anagen-specific expression of versican, in which the TSC1/TSC2/mTORC1 pathway may play a significant role.^64^

### Reprogrammed cells

The differentiation of stem cells into adult cells in response to defined factors is an important application of cell reprogramming. Induced pluripotent stem cells (iPSCs) have similar characteristics to embryonic stem cells in terms of morphology, self-renewal and differentiation capacity. They are not only free from ethical issues but also able to be propagated as autologous cells, which can avoid the complication of immune rejection. Thus, reprogramming of iPSCs into potential cells could be an approach to providing cell sources for HF regeneration. Under retinoic acid (RA), an iPSC-derived LNGFR^+^Thy1^+^ subpopulation with high proliferation could be induced into DPCs in the DP medium.^65^ The mixture of iPSC ectodermal precursor cells in keratinocyte culture medium with RA and bone morphogenetic protein (BMP) could also transform into new DPCs.^66^ Intriguingly, iPSCs could also be generated from human DPCs upon lentiviral transfection with Oct4, Sox2, Klf4 and c-Myc.^67^ In addition, iPSCs, post-culture on Matrigel, could differentiate into keratinocytes when treated with keratinocyte serum-free media supplemented with all-*trans* RA and BMP4.^68^ Human hair follicular keratinocytes could also be reprogrammed into iPSCs, and keratinocyte-derived iPSCs are further capable of differentiating into keratinocytes,^69^ which suggests a novel way to provide a source of keratinocytes. Lineage reprogramming is direct cellular reprogramming, which means that targeted cells could bypass the stem cell stage and convert directly to potential cells. Unlike traditional concepts regarding the epigenetic stability of differentiated cells, direct lineage reprogramming can transform one specialized cell type into another using defined factors, which is a more efficient and promising approach for producing functional cells. Treatment with the combination of FGF2, PDGF and 6-bromoindirubin-3′-oxime (BIO) could chemically transform human dermal fibroblasts into DPCs.^70^ SKPs can be produced from healthy adult fibroblasts via lineage reprogramming, and their cryopreservation can largely preserve their properties and produce more significant yields.^71^ Another way to rapidly expand SKPs via lineage reprogramming is to expose pre-established dermal fibroblasts to 30-min acid stress prior to isolating SKPs.^72^ Acute acidic stress treatment of dermal fibroblast cultures greatly improves SKP isolation, growth, yield and multipotency.

#### Overview

With progress in HF developmental biology and cellular reprogramming techniques, several cells with the potential for HF regeneration have been identified. These results have greatly expanded the seed cell bank for HF regeneration and solved the problem of the lack of a cell source. However, the problems these cell sources have in common are that their potential to regenerate HF fails to be maintained during long-term culture in vitro. In addition, HFSCs are few in number and extremely difficult to obtain, and SKPs in vitro senesce soon when isolated from their physiological environments.^73^ In addition, the efficiency of reprogramming by gene editing is still low. The optimization of the culture system in vitro and the improvement of reprogramming efficiency are challenges for HF regeneration. Therefore, we believe that the construction of 3D culture systems that simulate the in vivo environment may provide an alternative approach, similar to hydrogel scaffold-based cell culture, which contributes to maintaining cell proliferation and growth as well as the potential to regenerate HFs. In addition, chemical reprogramming, a new reprogramming technology, is characterized by high security and efficiency.^74^ To improve the reprogramming efficiency, the replacement of chemical reprogramming with gene editing has broad prospects in HF regeneration.

### Mechanisms for the restoration of HF-inducive capacity

After birth, mature and actively growing HFs eventually become anchored in the subcutis and then undergo hair cycling, periodically and spontaneously undergoing repetitive cycles of growth (anagen), apoptosis-driven regression (catagen) and relative quiescence (telogen). Therefore, the HF is regarded as a dynamic mini-organ. The activation and maintenance of hair cycling is a prerequisite for the functional regeneration of HFs. HFSCs play an indispensable role in maintaining hair cycling. The HFSC population remains largely quiescent during hair growth, but a subpopulation actively proliferates and promotes the production of the new hair shaft under the control of Axin2 expression.^75^ At the onset of anagen following stimulation by growth-inducing signals from the DP, primed HFSCs also contribute to new hair shaft production.^76^ Under induction by such signalling, primed HFSCs undergo rapid proliferation to fuel the initial stage of hair growth, and then, the quiescent HFSCs undergo a second round of activation that replenishes cells lost at the onset of anagen, finally supporting prolonged growth of the hair shaft.^56^ At the beginning of anagen, activated HFSCs migrate from the bulge to the matrix area and become transit-amplifying cells that proliferate and differentiate to form the new hair shaft.^77^ Therefore, the coordinated activation of primed HFSCs and quiescent HFSCs is instrumental for the maintenance of hair cycling. The mechanism underlying HFSC homeostasis and hair cycling regulation is a complex molecular controlling process (Table 1) that is highly dependent on hormonal action.^78^ WNT/β-catenin and BMP signalling are considered to be the core pathways in the regulation of hair cycling.^79^

**Table 1:** Molecular control mechanism of hair cycling regulation

Hair cycling phase	Molecular mechanism	Ref.
Anagen	Initiation: 1. Wnt/β-catenin pathway and BMP antagonists (e.g. Noggin) act as anagen-inducing signals 2. Expression of vitamin D receptor (VDR) on keratinocytes is essential for the maintenance of normal hair cycling, especially anagen initiation and keratinocyte stem cell function 3. Hairless, a corepressor of VDR, plays a central role in the hair cycle and controls hair fate decision via Wnt/β-catenin signalling. Progenitor keratinocytes from the bulge region differentiate into both epidermis and sebaceous glands. Progenitor keratinocytes fail to adopt the fate of hair keratinocytes in the mutant scalp due to the decreased Wnt/β-catenin signalling in the absence of the hairless protein 4. STAT5 activation acts as a mesenchymal switch to trigger natural anagen entry in post-developmental hair follicle cycling, and that activation in the DPCs begins in late catagen, reaches a peak in early anagen and disappears in the rest of the cycle 5. Mediator complex subunit 1 expressed in epidermal keratinocytes maintains the quiescence of keratinocytes, prevents the depletion of follicular stem cells and induces HFs to enter anagen normally 6. Hes1 is upregulated in the lower bulge/hair germ and modulates Shh responsiveness in anagen initiation, and there is a potential link between the Notch/Hes1 and Sonic Hedgehog pathways, in which Hes1 reinforces Hedgehog signalling at the onset of hair growth to expand the progenitors and replenish the stem cells to maintain hair cycle homeostasis 7. Leptin produced by human DPCs, as an anagen inducer, could stimulate keratinocytes to proliferate and participate in the control of the HF cycle through paracrine and autocrine mechanisms	^1,117–128^
	Maintenance: 1. Insulin-like growth factor-1 (IGF-1), hepatic growth factor and vascular endothelial growth factor are thought to be important for anagen maintenance, and the overexpression of isoform IGF-1 in keratinocytes accelerates HF formation and cycling in mice 2. Decorin promotes the proliferation and migration of ORS keratinocytes and maintains hair anagen in mice 3. Local injection of recombinant Hoxc13 polypeptide during the late anagen phase prolonged the anagen phase. RhHoxc13 injections during the telogen phase significantly promoted hair growth and induced anagen progression, and Hoxc13 could block anagen–catagen transition by inhibiting TGF-β1 signalling. 4. Artemis (Serine516) in mid-anagen and mature anagen was stronger than that in telogen and catagen, and it participates in the induced growth of mouse hair. 5. P-cadherin regulates human hair growth and anagen maintenance by regulating canonical Wnt signalling and suppressing TGF-β2	^1,129–135^
Anagen–catagen transition	1. IGF-1 signalling acts on the anagen-to-catagen transition in the hair cycle, partly through BMP4 activation 2. VDR, the transcriptional repressor hairless and the RA receptor control anagen–catagen transformation, and hairless interacts with VDR to regulate transcription in mice. In the absence of these regulators, HFs disintegrate into epithelial sacs and dermal cysts upon catagen entry 3. Peripheral core clock genes (CLOCK, BMAL1 and Period1) are an integral component of the human hair cycle clock and are involved in the anagen-to-catagen transition 4. TNF-α is required for timely anagen–catagen transition in mouse pelage follicles, and its ablation partially rescues the hair cycling defect of K17-null mice	^1,119,129,131,136–140^
Catagen	1. FGF5-deficient mice have blocked catagen entry and a prolonged anagen phase 2. The epidermal growth factor receptor triggers catagen entry in mice 3. TGF-β1, interleukin-1β, neurotrophins (NT-3, NT-4 and BDNF), BMP2/4 and TNF-α have also been reported to induce catagen 4. Inhibition of heparanase (a heparan sulfate endoglycosidase) in HFs cultured in vitro has been shown to induce a catagen-like process, and heparan sulfate regulates hair follicle and sebaceous gland morphogenesis and homeostasis 5. Intrinsic Wnt7b expression regulates HFSC homeostasis and controls anagen length and catagen entry	^1,117,129,141–145^
Catagen–telogen transition	GasderminA3 controls the catagen–telogen transition by balancing the Wnt signalling pathway	^146^
Telogen	1. CCN2, restricted to the dermal papillae and ORS, normally acts to maintain stem cell quiescence. The deletion of CCN2 results in a shortened telogen phase length and an elevated number of HF 2. Cyclic dermal BMP signalling regulates stem cell activation during hair regeneration. The overexpression of BMP antagonist (noggin) in mouse skin resulted in a markedly shortened refractory phase and faster propagation of the regenerative wave	^27,147^
Telogen–anagen transition	1. The balance of BMP6 and Wnt10b regulates the telogen–anagen transition of hair follicles, and they regulate HFSCs activation competitively 2. Endoglin, a TGFβ/BMP co-receptor, could maintain a correct follicle cycling pattern and adequate stimulation of the HFSC niche. The telogen–anagen switching mechanism is based on Eng-dependent feedback crosstalk between the Wnt/β-catenin and Bmp/Smad signals 3. NF-κB participates in the telogen–anagen transition in awl and zigzag HFs and is also required for zigzag hair bending and HF cycling	^148–150^

#### Overview

The dynamic characteristics of HFs enable their sustainable and periodic regeneration. We think that the activation and maintenance of hair cycling are indispensable for achieving functional HF regeneration. Past studies have not only led to a better understanding of the molecular mechanisms of hair cycling, which makes it more possible to find the key molecules therein, but also have revealed the complexity of HF dynamic characteristics. Therefore, it is still difficult to discover the key molecular event in hair cycling.

## In vivo strategies for functional regeneration of HFs

### Cell transplantation and HF regeneration

Cell-based transplantation without biomaterials is a minimally invasive approach to in vivo HF regeneration. Current cell transplantation mainly involves the transplantation of stem cells or a mixture of epidermal and dermal components. The injection of a mixture containing Epi-SCs and DPCs into nude mice could induce new HFs with the correct histological structures and form a multilayered stratified epidermis containing HF-like structures.^20^ Although the new HFs are relatively small, this result proves again that the rearrangement of the EMI and the niches of the potential cells are essential and necessary for HF construction. A mixture of Epi-SCs and SKPs was grafted into excisional wounds in nude mice, and a bilayer structure resembling the epidermis and the dermis formed on the fifth day, followed by de novo HF.^80^ In the regeneration process, the SKPs formed DPCs in neogenic HFs and abundant dermal cells in the dermis, and the Epi-SCs formed the epidermis and trunk of the HF. More importantly, this experiment also demonstrates that the PI3K-Akt signalling pathway plays a crucial role in the interactions between Epi-SCs and SKPs and de novo HF regeneration, which may suggest potential therapeutic applications in enhancing hair regeneration.^80^ During the hair growth cycle, HFSCs periodically switch between the active and inactive stages to maintain stem cell populations and generate new HFs, while this potential is impaired in aged HFSCs.^81^ However, in transplantation assays in vivo, aged HFSCs could still regenerate HFs when supported with the young dermis, while young HFSCs failed to regenerate HFs when combined with the aged dermis, which shows that the ageing skin microenvironment dictates stem cell behaviour and illustrates the dominant role of the niche microenvironment.^61^ This research discovered that the HF regeneration potential of aged HFSCs can be rejuvenated by neonatal dermis during in vivo transplantation, providing promising new avenues for regenerative and geriatric medicine. In recent years, for the fully functional regeneration of ectodermal organs, a bioengineered organ germ has been developed by reproducing the embryonic processes of organogenesis, including bioengineered HFs.^82^ Bioengineered HF germ could be reconstituted with embryonic skin-derived epithelial and mesenchymal cells and was able to develop histologically correct HFs when ectopically transplanted. The bioengineered HFs not only properly connected to the host skin epithelium by intracutaneous transplantation and reproduced the stem cell niche and hair cycling but also autonomously connected with nerves and the arrector pili muscle at the permanent region and exhibited piloerection ability.^83^ Another way to construct bioengineered HF is with pelage and vibrissae reconstituted with embryonic skin-derived cells and adult vibrissa stem cells. After intracutaneous transplantation, this bioengineered HF germ not only develops the correct structures and forms proper connections with surrounding host tissues such as the epidermis, arrector pili muscle and nerve fibres but also shows restored hair cycling and piloerection through the rearrangement of follicular stem cells and their niches, with fully functional hair organ regeneration.^84^ It is worth mentioning that the in vivo transplantation of bioengineered HFs also achieves the partial restoration of hair cycling, which is a great step forward for functional HF regeneration.

### Cellular reprogramming and HF regeneration

Cellular reprogramming is not only a tool for tissue engineering to enrich potential cell sources for the regeneration of HF but also a participant in physiological de novo HF induction. Secreted proteins (apolipoprotein-A1, galectin-1 and lumican) from embryonic skin conferred upon non-hair fibroblasts the competency to regenerate HF via the activation of IGF and WNT signalling, thereby endowing non-HF skin with the ability to reproduce HFs, which suggests the involvement of cellular reprogramming.^85^ Because DPCs and dermal fibroblasts originate from common fibroblast progenitors in the developing embryonic mouse skin and have highly correlated gene expression profiles (96%),^86^ adult dermal fibroblasts can be reprogrammed into a neonatal state, with the capacity of inducing ectopic HF formation similar to DPCs through the epidermal activation of β-catenin.^87^ Furthermore, treatment with the combination of FGF2, PDGF and BIO in adherent culture followed by suspension culture could induce the generation of DP-like cells from foetus- or adult foreskin-derived fibroblasts. The integration of foetal/adult DP-like cells can be recruited to replenish DP of de novo generated HFs, and the regenerated HF structures were reconstructed in 65% nude mice implanted with foetal DP-like cells and in 70% nude mice with adult DP-like cells.^70^ Finally, the combination of MITF, SOX10 and PAX3 could directly convert mouse and human fibroblasts into induced melanocytes, which have the ability to generate pigmented epidermis and HF in vivo when properly integrated into the dermal–epidermal junction.^88^ Healed wounds with the loss of HF are usually filled with a large number of fibroblasts, so the exploration of fibroblast-based reprogramming holds great promise for HF regeneration in situ.

### Cell-laden biomaterials for the establishment of HF equivalents

Biomaterials are implantable, inactive materials that can replace or repair damaged tissue with high biocompatibility. Biomaterials can create a 3D environment for cell-to-cell interactions, simulating the function of cell niches to a certain extent, and they have been widely used in wound repair and tissue regeneration.^89^ The combination of potential cells with biomaterials, such as hydrogels, scaffolds and other self-assembled materials, could contribute to HF regeneration (Figs. 1c and 2). For in vivo HF regeneration, a 3D hydrogel with a mixture of epidermal keratinocytes, dermal cells and β-catenin-expressing CD133^+^ DPCs not only performed better than CD133^+^ DPCs alone (average of 28 ± 6 HF per field vs. 13 ± 6 HF per field) but also exhibited a more advanced hair cycling stage;^90^ an average of 71% of the HFs reached anagen stage III, and 19% reached anagen stage IV in reconstituted skin containing β-catenin-expressing CD133^+^ DPCs, while 67.5% of the HFs in the control remained in anagen I and II, and only 27.5% reached anagen III.^90^ The stable expression of β-catenin could promote the clonal growth of CD133^+^ DPCs in vitro in 3D hydrogel culture. An alternative strategy to reproduce EMI is to use a collagen-chitosan scaffold (CCS)-based 3D system containing dissociated epithelial cells and DPCs followed by treatment of the CCS with Wnt-CM from Wnt1a^+^ bone marrow mesenchymal stem cells. The results suggested that the cell mixture was able to induce hair regeneration in nude mice, and Wnt-CM can maintain the hair induction ability of DPCs in expansion cultures, which may be associated with the activation of the Wnt/β-catenin signalling pathway.^91^ Therefore, how to enhance the HF induction efficiency of cultured human DPCs is a priority in bioengineering for clinical HF regeneration. It has been reported that DPCs retain HF inductivity best when cultured and transplanted as multicellular aggregates, and DP spheroids could form a structure similar to the natural intercellular organization in vivo.^92^ Therefore, another strategy is to achieve the scalable fabrication of controllable DP spheroids to regenerate HFs. Scalable production of controllable DP spheroids on polyvinyl alcohol surfaces has high viability and preserves DP characteristics, and at DPC numbers of 5 × 10^3^ to 30 × 10^3^ cells each, both human and rat DP spheroids are able to induce HF neogenesis. Larger DP spheroids exhibit higher HF inductivity. The researchers developed a method that can be automated for mass production of DP spheroids with controllable size and cell number in a wide range,^93^ although the average diameter of regenerated hair fibre did not significantly change with increasing size of the transplanted DP spheroids. Likewise, the hanging-drop approach could also lead to a controllable 3D spheroid model for the scalable fabrication of inductive DP microtissues. That technique is based on surface tension and the interaction between surface tension and a gravity field that causes the convergence of liquid drops. With the converged drops, DP spheroids could endow high-passaged DP microtissues with many similarities to primary DP. Subcutaneous implantation of these microtissues mixed with new-born mouse epidermal cells has achieved reproducible HF induction in the hypodermis of nude mice, and a large amount of extracellular matrix (ECM) components is found in the intercellular space within the DP microtissue, similar to an anagen DP.^94^ These models provide the potential to elucidate the native biology of human DP and show promise for the controllable and scalable production of inducive DPCs for future follicle regeneration. Exosomes derived from DP spheroids (3D DP-Exos) are also able to promote the proliferation of DPCs and ORS cells and accelerate anagen onset to influence hair cycling. Local injections of 3D DP-Exos (exosomes) could induce anagen from telogen and prolong anagen in mice. Moreover, DPC spheres treated with Exos could augment HF neogenesis when implanted with mouse epidermal cells,^95^ which may be associated with high levels of miR-218-5p in Exos.^96^ Cell surface engineering technology also advances HF regeneration by accurate micro/nanoscale control in cell-biomaterial ensembles and DP spheroid formation. Owing to the security and tuneable thickness at the nanoscale, the nanogel could encapsulate a single cell by layer-by-layer (LbL) self-assembly and further form DPC spheroids by physical cross-linking on nanogel-coated cells. LbL-DPC aggregation is akin to that of primary DPCs and has the capacity to restore HF induction potential in vitro and regenerate HFs in vivo.^97^ Other biomaterials that contribute to the restoration of HF-inducive ability include human placenta ECM hydrogel,^98^ synthesized ECM^99^ and a chitosan/polyvinyl alcohol nanofibre sponge with an open-cell cellular structure,^100^ which expands the biomaterial libraries for the optimization of the culture environment in vitro to restore the ability of DPCs to regenerate HFs. 3D printing technology for HF regeneration by recapitulating the physiological 3D organization of cells in the HF microenvironment is an innovative biomimetic approach.^101^ It permits the controllable self-aggregating spheroid formation of DPCs in a physiologically relevant ECM, the initiation of EMI and further HF formation in human skin constructs. Remarkably, the vascularization of HF-bearing human skin constructs increases graft survival and enables efficient human hair growth in mice.^101^ This method represents a novel bioengineering strategy for the feasible generation of hair-bearing human skin constructs entirely ex vivo from cultured human cells, and adaptation of this new technology by hair researchers, hair restoration surgeons and the pharmaceutical industries will have overwhelming implications in the maintenance and regeneration of HFs.

Similar to DPCs, long-term culture could also impair the HF-inducive capacity of SKPs. It is likely that SKPs rely on special environments for their self-renewal and stable gene expression. In tissue engineering, scaffolds are created to mimic environments for stem cells to survive, differentiate and form functional tissue structures. In this regard, scaffolds such as hydrogels and matrix could be candidates to support stem cells for organogenesis and regenerate HFs.^102,103^ Self-assembling peptide nanofibres are made of natural amino acids and form hydrogels that surround cells in a manner similar to the ECM. RADA16 (Ac-(RADA)4-CONH2) is a representative example of such peptides that has been shown to promote nerve regeneration and wound healing.^104,105^ A PRG (PRGDSGYRGDS) solution functionalized by mixing with RADA16 promoted the proliferation and migration of periodontal ligament fibroblasts.^106^ The self-assembling peptide hydrogels formed by RADA16 and PRG (RADA-PRG) could facilitate the attachment, proliferation and survival of SKPs, ultimately supporting HF neogenesis in vivo. The transplantation of a combination of culture-expanded SKPs and neonatal epidermal cells into RADA-PRG hydrogel resulted in a significantly increased number of neogenic hairs compared to Matrigel and other peptide hydrogels. This may be attributed to the similarity of the properties of these designer peptide nanofibres to those of ECM molecules.^102^ In addition, a mixture of culture-expanded Epi-SC and SKPs derived from the adult human scalp in a hydrogel was capable of reconstituting functional HFs, and the mechanisms of the expression of ALP in SKPs in vitro and the maintenance of HF-inducive properties in vivo may be associated with BMP4.^103^ More importantly, Epi-SC implanted in wounds in combination with SKPs could also form functional sebaceous glands in association with HFs. Normal human neonatal foreskin keratinocytes were induced to differentiate into several cellular components that compose normal HFs, with the expression of anagen-specific versican when grown on a collagen matrix embedded with TSC2-null fibroblast-like cells or with fibroblasts.^64^ Regenerated HFs were complete with sebaceous glands, hair shafts and inner and ORSs.^64^

### Drug delivery systems for HF construction

Drug delivery systems consist of molecules with pharmacological activity modified into advanced materials, which have been widely used in skin wound treatment. Currently, it has been reported that many drug delivery systems could promote wound healing as well as HF regeneration. Newly developed multidomain peptide hydrogels have exhibited regenerative potential in a diabetic wound healing model, resulting in wound closure, accelerated HF regeneration and a greater average number of HFs at both the edge and the centre.^107^ Multifunctional Zn-doped hollow mesoporous silica/polycaprolactone electrospun membranes exhibit excellent antibacterial activity for wound healing and are ~20 times as likely to regenerate HFs as the control.^108^ Most excitingly, the treatment of deep second-degree scalding injuries with human erythropoietin (EPO) to, whether by the local subcutaneous injection of nanosized rhEPO (recombinant human EPO)/infusion pumping or the topical application of rhEPO gel, achieved excellent skin repair with conical and HF structures, which was related to the combined expression of EPO receptor and β-subunit receptor.^109,110^ A novel fibrous membrane (P/Qu/Cup, P: PCL, Qu: Quercetin, Cup: cuprorivaite, CaCuSi_4_O_10_) containing quercetin-copper (Qu-Cu) chelates was fabricated by using quercetin and highly bioactive bioceramic (CaCuSi_4_O_10_) incorporated in PCL/gelatine electrospun fibres. The fibrous membrane can effectively release Qu and Cu ions to induce the proliferation, migration and differentiation of skin- and HF-related cells, and the Qu, Cu ions and Si ions released from the composite membrane revealed synergistic activity to stimulate HF regeneration and wound healing in burned skin.^111^ However, drug delivery systems mainly contribute to wound repair, accompanied by the acceleration of wound-induced HF neogenesis (WIHN). WIHN is a regenerative phenomenon separate from physiological regeneration, as its cellular origin is not from the HFSCs in the bulge at the wound edge. In WIHN, a fully functional follicle can regenerate in the centre of a full-thickness wound with a large enough size, and the cellular origin of this process is similar to an embryonic process. The neogenic follicles have similar functions to embryonic HFs, which also have a growth cycle.^63^ Currently, there are few studies about drug delivery systems for HF regeneration alone outside the wound environment. However, because of their controllability, targeted delivery, sustained release and even intellectuality, we think drug delivery systems hold great promise for HF regeneration in the future.

### Organoid technology and HF replacement

An organoid is defined as a 3D structure grown from organ-specific stem cell types. It can recapitulate key aspects of in vivo organs and avoid many of the disadvantages associated with cell lines.^112^ HF organoids can be established from skin stem cells or a mixture of dermal and epidermal components. DP spheroids encapsulated by silk-gelatine hydrogel and HF keratinocytes as well as stem cells could be used to construct in vitro HF organoids. These organoids show enhanced DPC-specific gene expression and ECM production, and their structural features and cell–cell interactions are similar to those of in vivo HFs.^113^ This simple in vitro DP organoid model system has the potential to provide significant insights into the underlying mechanisms of HF morphogenesis and distinct molecular signals relevant to different stages of the hair cycle and hence can be used for the controlled evaluation of the efficacy of new drug molecules to induce HF regeneration. To date, in vitro skin derivation strategies have focused on first generating keratinocytes and fibroblasts from iPSCs in separate cultures and then combining the two types of cells to form a skin-like bilayer. A major challenge is how to realize the synchronous construction of its appendages. Under initial treatment with the TGF-β inhibitor SB431542 (SB) and recombinant BMP4 and subsequent treatment with FGF2 (FGF) and a BMP inhibitor LDN-193189 (LDN), a homogeneous population of mouse pluripotent stem cells and constituting epidermal and dermal layers in a 3D culture formed skin organoids with a cyst conformation, in which HFs were spontaneously produced de novo. However, the new HFs entered catagen and degenerated during long-term culture.^114^ These results suggest how skin organoid structures can be generated de novo without the use of embryonic tissue or undefined media, which will be useful for studying the minimal cellular and microenvironment requirements for HF induction. Scalp-derived dermal progenitor cells mixed with foreskin-derived Epi-SCs at a 2:1 ratio could aggregate in suspension to form a large number of HF organoids, and the dermal and epidermal cells self-assembled into distinct epidermal and dermal compartments. The addition of recombinant WNT3a protein to the medium enhanced the formation of these aggregates, and the transplantation of these organoids in vivo achieved HF formation.^115^ Finally, a 3D integumentary organ system (IOS) obtained by the self-assembly of mesenchymal and Epi-SCs from iPSCs using the clustering-dependent embryoid body transplantation method also regenerated fully functional HF organoids.^116^ After transplantation into nude mice, the HF organoid in that system could form proper connections to the surrounding host tissues, such as the epidermis, arrector pili muscles and nerve fibres, without tumorigenesis, and show appropriate hair eruption and hair cycles, including the rearrangement of follicular stem cells and their niches.^116^ These findings reveal the generation of a bioengineered 3D IOS from iPSCs, including appendage organs such as HFs and sebaceous glands, with appropriate connections to surrounding tissues, which significantly advances the technological development of the bioengineered 3D IOS and its potential applications, including an in vitro assay system, an animal model alternative and bioengineered organ replacement therapy.

#### Overview

The transition from traditional culture to 3D culture constitutes excellent progress in HF regeneration. 3D culture enhances the proliferation and HF regeneration ability of potential cells, and a mixture of epidermal (Epi-SC) and dermal (SKP and DPCs) components in a 3D system simulates the characteristics of EMI, especially organoid technology and 3D printing technology. In 3D culture, regenerated HFs in vivo not only connect appropriately to surrounding host tissues but also undergo hair cycling activation. However, some problems are less thoroughly addressed. How long can the regenerated HFs last? Can the regenerated HFs go through full hair cycling? Will HF regeneration-induced hair be superior to transplanted hair? We think these are key questions and important challenges in functional HF regeneration.

### Achievements, limitations and future perspectives

Much progress has been made in the developmental biology and regeneration of HFs. (1) A variety of cells with the potential to regenerate HFs have been identified, including DPCs, SKPs, HFSCs, keratinocytes and reprogrammed cells (iPSCs and fibroblasts), which provide a wide range of cell sources for HF regeneration. (2) We have gained a more comprehensive and in-depth understanding of the hair cycling-related mechanism, which provides the biological basis for finding key molecules to initiate and sustain the hair cycle. (3) Optimization of the in vitro culture system and the construction of a 3D culture environment could overcome the loss of the ability of potential cells to proliferate, self-renew and regenerate HFs caused by 2D culture. (4) The transplantation of a mixture of epidermal and dermal components, such as cell-based transplantation with or without biomaterials and HF organoids, could simulate EMI to a certain extent and successfully induce new HFs with the correct structure in vivo. (5) The HF organoid is a model for the exploration of mechanisms of HF morphogenesis and drug research to reproduce hair. (6) Drug delivery systems are characterized by strong controllability and high security, and they can promote wound healing accompanied by HF regeneration.

Since it is an architecturally and functionally complex organ, the HF is much more difficult to regenerate or reconstruct than many other organs. Due to this limitation, HF regeneration is still far from clinical transformation. (1) The sources of potential cells are still poor, largely because of cell ageing in vitro culture and inefficient reprogramming, so there is still a need to optimize the in vitro culture system. (2) The mechanism of hair cycling is very complex, and it is extremely difficult to identify the key molecules. (3) Current strategies simulating EMI are still insufficient. Both cell transplantation and organoid architecture lack the microenvironment of connective tissue, blood vessels and immune cells, which is still quite different from the physiological environment of normal tissues and organs. (4) It is unknown how many new HFs can be regenerated from biomaterials and tissue engineering. Do they allow other essential cells to be recruited to the new follicle? If so, do the attracted cells have the ability to affect organogenesis overall? 5) The cellular reprogramming techniques that contribute to HF regeneration still have low efficiency in vitro. (6) The 3D regeneration of HFs depends on biomaterials that need better external security, controllability and internal stability. Ideal biomaterials need to be safe and nontoxic. Under normal metabolism in the body, they can be kept in a stable state without biological degeneration, and the metabolism or degradation products are harmless and easily metabolized. (7) Whether the regenerated HFs function normally and how long they can last in vivo are less mentioned in past studies.

In summary, at the current stage, various attempts are only imitating a partial structure and/or function regeneration of HFs. The combination of different technologies and methodologies will hopefully lead to new progress. For example, the creation of transplantable HFs that closely mimic the structures and functions of native tissue may be accomplished by combining organoid technology with a drug delivery system. Depending on the controllable release of the relevant factors in hair cycling, such as WNT or BMP, hair cycling activation and maintenance of HF organs may be achieved. We also need to continue to optimize the in vitro culture systems of potential cells and look for more efficient reprogramming techniques, such as chemical reprogramming induced by small molecules or genetic reprogramming of genes delivered by biomaterials. Finally, we want to reiterate that, based on existing work, it is worth considering whether the achievement of the activation and maintenance of hair cycling in regenerated HFs could be the heart of the next phase.

## References

[1] Paus R, Foitzik K (2004). In search of the “hair cycle clock”: a guided tour. Differentiation.

[2] Rezza A (2016). Signaling networks among stem cell precursors, transit-amplifying progenitors, and their niche in developing hair follicles. Cell Rep..

[3] Ren X (2020). Lgr4 deletion delays the hair cycle and inhibits the activation of hair follicle stem cells. J. Invest. Dermatol..

[4] Houschyar KS (2020). Molecular mechanisms of hair growth and regeneration: current understanding and novel paradigms. Dermatology.

[5] Talavera-Adame D, Newman D, Newman N (2017). Conventional and novel stem cell based therapies for androgenic alopecia. Stem Cells Cloning.

[6] Jahoda CA, Whitehouse J, Reynolds AJ, Hole N (2003). Hair follicle dermal cells differentiate into adipogenic and osteogenic lineages. Exp. Dermatol..

[7] Driskell RR (2013). Distinct fibroblast lineages determine dermal architecture in skin development and repair. Nature.

[8] Millar SE (2002). Molecular mechanisms regulating hair follicle development. J. Invest. Dermatol..

[9] Horne KA, Jahoda CA, Oliver RF (1986). Whisker growth induced by implantation of cultured vibrissa dermal papilla cells in the adult rat. J. Embryol. Exp. Morphol..

[10] Jahoda CA (1996). Human hair follicle regeneration following amputation and grafting into the nude mouse. J. Invest. Dermatol..

[11] Kishimoto J, Burgeson RE, Morgan BA (2000). Wnt signaling maintains the hair-inducing activity of the dermal papilla. Genes Dev..

[12] Jahoda CA, Horne KA, Oliver RF (1984). Induction of hair growth by implantation of cultured dermal papilla cells. Nature.

[13] Ferraris C, Bernard BA, Dhouailly D (1997). Adult epidermal keratinocytes are endowed with pilosebaceous forming abilities. Int. J. Dev. Biol..

[14] Blanpain C (2004). Self-renewal, multipotency, and the existence of two cell populations within an epithelial stem cell niche. Cell.

[15] Xing L, Kobayashi K (2001). Ability of transplanted cultured epithelium to respond to dermal papillae. Tissue Eng..

[16] Ehama R (2007). Hair follicle regeneration using grafted rodent and human cells. J. Invest. Dermatol..

[17] Zhang L (2019). Induction of hair follicle neogenesis with cultured mouse dermal papilla cells in de novo regenerated skin tissues. J. Tissue Eng. Regen. Med..

[18] Ferraris C, Chaloin-Dufau C, Dhouailly D (1994). Transdifferentiation of embryonic and postnatal rabbit corneal epithelial cells. Differentiation.

[19] Fliniaux I, Viallet JP, Dhouailly D, Jahoda CAB (2004). Transformation of amnion epithelium into skin and hair folliclesregeneration. Differentiation.

[20] Zhang M (2020). Preliminary studies of hair follicle regeneration by injections of epidermal stem cells and dermal papilla cells into nude mice. Cell Tissue Bank.

[21] Zhou L (2016). CD133-positive dermal papilla-derived Wnt ligands regulate postnatal hair growth. Biochem. J..

[22] Feng M, Yang G, Wu J (2011). Versican targeting by RNA interference suppresses aggregative growth of dermal papilla cells. Clin. Exp. Dermatol..

[23] Matsuzaki T, Yoshizato K (1998). Role of hair papilla cells on induction and regeneration processes of hair follicles. Wound Repair Regen..

[24] Lu ZF (2004). Expressions of bFGF, ET-1 and SCF in dermal papilla cells and the relation to their biological properties. Zhejiang Da Xue Xue Bao Yi Xue Ban..

[25] Trueb RM (2018). Further clinical evidence for the effect of IGF-1 on hair growth and alopecia. Ski. Appendage Disord..

[26] Na JI (2012). Histidine decarboxylase expression influences the neofolliculogenesis of newborn mouse dermal cells. J. Dermatol. Sci..

[27] Liu S, Leask A (2013). CCN2 modulates hair follicle cycling in mice. Mol. Biol. Cell.

[28] Lim CH (2018). Hedgehog stimulates hair follicle neogenesis by creating inductive dermis during murine skin wound healing. Nat. Commun..

[29] Yu Z (2018). Hoxc-dependent mesenchymal niche heterogeneity drives regional hair follicle regeneration. Cell Stem Cell.

[30] Lee YR (2019). Monoterpenoid loliolide regulates hair follicle inductivity of human dermal papilla cells by activating the Akt/beta-catenin signaling pathway. J. Microbiol. Biotechnol..

[31] Reynolds AJ (1999). Trans-gender induction of hair follicles. Nature.

[32] Kang BM (2012). Sphere formation increases the ability of cultured human dermal papilla cells to induce hair follicles from mouse epidermal cells in a reconstitution assay. J. Invest. Dermatol..

[33] Choi M (2020). Glucose metabolism regulates expression of hair-inductive genes of dermal papilla spheres via histone acetylation. Sci. Rep..

[34] Lin, B. J. et al. LncRNA-XIST promotes dermal papilla induced hair follicle regeneration by targeting miR-424 to activate hedgehog signaling. *Cell Signal.* 109623 (2020). 10.1016/j.cellsig.2020.109623 32243962

[35] Ahmed NS (2017). Epidermal E-cadherin dependent beta-catenin pathway is phytochemical inducible and accelerates anagen hair cycling. Mol. Ther..

[36] Aoi N (2012). 1Alpha,25-dihydroxyvitamin D3 modulates the hair-inductive capacity of dermal papilla cells: therapeutic potential for hair regeneration. Stem Cells Transl. Med..

[37] Miao Y (2013). Effect of PRP on the proliferation of dermal papilla cells and hair follicle regeneration in mice. Zhonghua Zheng Xing Wai Ke Za Zhi..

[38] Shen H, Cheng H, Chen H, Zhang J (2017). Identification of key genes induced by platelet-rich plasma in human dermal papilla cells using bioinformatics methods. Mol. Med. Rep..

[39] Su YS (2017). Icariin promotes mouse hair follicle growth by increasing insulin-like growth factor 1 expression in dermal papillary cells. Clin. Exp. Dermatol..

[40] Inamatsu M, Matsuzaki T, Iwanari H, Yoshizato K (1998). Establishment of rat dermal papilla cell lines that sustain the potency to induce hair follicles from afollicular skin. J. Invest. Dermatol..

[41] Rheinwald JG, Green H (1975). Serial cultivation of strains of human epidermal keratinocytes: the formation of keratinizing colonies from single cells. Cell.

[42] Rendl M, Polak L, Fuchs E (2008). BMP signaling in dermal papilla cells is required for their hair follicle-inductive properties. Genes Dev..

[43] Osada A (2007). Long-term culture of mouse vibrissal dermal papilla cells and de novo hair follicle induction. Tissue Eng..

[44] Harel S (2015). Pharmacologic inhibition of JAK-STAT signaling promotes hair growth. Sci. Adv..

[45] Toma JG (2001). Isolation of multipotent adult stem cells from the dermis of mammalian skin. Nat. Cell Biol..

[46] Biernaskie J (2009). SKPs derive from hair follicle precursors and exhibit properties of adult dermal stem cells. Cell Stem Cell.

[47] Fernandes KJ (2004). A dermal niche for multipotent adult skin-derived precursor cells. Nat. Cell Biol..

[48] Rahmani W (2014). Hair follicle dermal stem cells regenerate the dermal sheath, repopulate the dermal papilla, and modulate hair type. Dev. Cell.

[49] Guo L (2019). TSA restores hair follicle-inductive capacity of skin-derived precursors. Sci. Rep..

[50] Gonzalez R (2017). Platelet-derived growth factor signaling modulates adult hair follicle dermal stem cell maintenance and self-renewal. NPJ Regen. Med..

[51] Hagner A, Biernaskie J (2013). Isolation and differentiation of hair follicle-derived dermal precursors. Methods Mol. Biol..

[52] Wang X, Dong S, Wu Y (2019). Isolation and cultivation of skin-derived precursors. Methods Mol. Biol..

[53] Agabalyan NA (2017). Enhanced expansion and sustained inductive function of skin-derived precursor cells in computer-controlled stirred suspension bioreactors. Stem Cells Transl. Med..

[54] Ohyama M (2007). Hair follicle bulge: a fascinating reservoir of epithelial stem cells. J. Dermatol. Sci..

[55] Hsu YC, Li L, Fuchs E (2014). Transit-amplifying cells orchestrate stem cell activity and tissue regeneration. Cell.

[56] Greco V (2009). A two-step mechanism for stem cell activation during hair regeneration. Cell Stem Cell.

[57] Hsu YC, Pasolli HA, Fuchs E (2011). Dynamics between stem cells, niche, and progeny in the hair follicle. Cell.

[58] Rhee H, Polak L, Fuchs E (2006). Lhx2 maintains stem cell character in hair follicles. Science.

[59] Vidal VP (2005). Sox9 is essential for outer root sheath differentiation and the formation of the hair stem cell compartment. Curr. Biol..

[60] Zhang S (2012). Hair follicle stem cells derived from single rat vibrissa via organ culture reconstitute hair follicles in vivo. Cell Transplant..

[61] Ge Y (2020). The aging skin microenvironment dictates stem cell behavior. Proc. Natl Acad. Sci. USA.

[62] Beaudoin GM, Sisk JM, Coulombe PA, Thompson CC (2005). Hairless triggers reactivation of hair growth by promoting Wnt signaling. Proc. Natl Acad. Sci. USA.

[63] Ito M (2007). Wnt-dependent de novo hair follicle regeneration in adult mouse skin after wounding. Nature.

[64] Li S (2011). Human TSC2-null fibroblast-like cells induce hair follicle neogenesis and hamartoma morphogenesis. Nat. Commun..

[65] Ohyama M (2012). Restoration of the intrinsic properties of human dermal papilla in vitro. J. Cell Sci..

[66] Veraitch O (2013). Human induced pluripotent stem cell-derived ectodermal precursor cells contribute to hair follicle morphogenesis in vivo. J. Invest. Dermatol..

[67] Muchkaeva IA (2014). Generation of iPS cells from human hair follice dermal papilla cells. Acta Nat..

[68] Itoh M, Kiuru M, Cairo MS, Christiano AM (2011). Generation of keratinocytes from normal and recessive dystrophic epidermolysis bullosa-induced pluripotent stem cells. Proc. Natl Acad. Sci. USA.

[69] Lim SJ (2016). Induced pluripotent stem cells from human hair follicle keratinocytes as a potential source for in vitro hair follicle cloning. PeerJ.

[70] Zhao Q (2019). Chemically induced transformation of human dermal fibroblasts to hair-inducing dermal papilla-like cells. Cell Prolif..

[71] Wenzel V (2012). Naive adult stem cells from patients with Hutchinson-Gilford progeria syndrome express low levels of progerin in vivo. Biol. Open.

[72] Budel L, Djabali K (2017). Rapid isolation and expansion of skin-derived precursor cells from human primary fibroblast cultures. Biol. Open.

[73] Liu S (2011). The PI3K-Akt pathway inhibits senescence and promotes self-renewal of human skin-derived precursors in vitro. Aging Cell.

[74] Hou P (2013). Pluripotent stem cells induced from mouse somatic cells by small-molecule compounds. Science.

[75] Lim X (2016). Axin2 marks quiescent hair follicle bulge stem cells that are maintained by autocrine Wnt/beta-catenin signaling. Proc. Natl Acad. Sci. USA.

[76] Ito M, Kizawa K, Hamada K, Cotsarelis G (2004). Hair follicle stem cells in the lower bulge form the secondary germ, a biochemically distinct but functionally equivalent progenitor cell population, at the termination of catagen. Differentiation.

[77] Taylor G (2000). Involvement of follicular stem cells in forming not only the follicle but also the epidermis. Cell.

[78] Paus R (2014). Neuroendocrinology of the hair follicle: principles and clinical perspectives. Trends Mol. Med..

[79] Schneider MR, Schmidt-Ullrich R, Paus R (2009). The hair follicle as a dynamic miniorgan. Curr. Biol..

[80] Chen Y (2020). PI3K/Akt signaling pathway is essential for de novo hair follicle regeneration. Stem Cell Res. Ther..

[81] Matsumura H (2016). Hair follicle aging is driven by transepidermal elimination of stem cells via COL17A1 proteolysis. Science.

[82] Nakao K (2007). The development of a bioengineered organ germ method. Nat. Methods.

[83] Asakawa K (2012). Hair organ regeneration via the bioengineered hair follicular unit transplantation. Sci. Rep..

[84] Toyoshima KE (2012). Fully functional hair follicle regeneration through the rearrangement of stem cells and their niches. Nat. Commun..

[85] Fan SM (2018). Inducing hair follicle neogenesis with secreted proteins enriched in embryonic skin. Biomaterials.

[86] Morgan BA (2014). The dermal papilla: an instructive niche for epithelial stem and progenitor cells in development and regeneration of the hair follicle. Cold Spring Harb. Perspect. Med..

[87] Collins CA, Kretzschmar K, Watt FM (2011). Reprogramming adult dermis to a neonatal state through epidermal activation of beta-catenin. Development.

[88] Yang R (2014). Direct conversion of mouse and human fibroblasts to functional melanocytes by defined factors. Nat. Commun..

[89] Kim HS (2019). Advanced drug delivery systems and artificial skin grafts for skin wound healing. Adv. Drug Deliv. Rev..

[90] Zhou L (2016). Activating beta-catenin signaling in CD133-positive dermal papilla cells increases hair inductivity. FEBS J..

[91] Dong L (2017). Wnt1a maintains characteristics of dermal papilla cells that induce mouse hair regeneration in a 3D preculture system. J. Tissue Eng. Regen. Med..

[92] Young TH (2008). Self-assembly of dermal papilla cells into inductive spheroidal microtissues on poly(ethylene-co-vinyl alcohol) membranes for hair follicle regeneration. Biomaterials.

[93] Huang YC (2013). Scalable production of controllable dermal papilla spheroids on PVA surfaces and the effects of spheroid size on hair follicle regeneration. Biomaterials.

[94] Lin B (2016). Surface tension guided hanging-drop: producing controllable 3D spheroid of high-passaged human dermal papilla cells and forming inductive microtissues for hair-follicle regeneration. ACS Appl. Mater. Interfaces.

[95] Kwack MH (2019). Exosomes derived from human dermal papilla cells promote hair growth in cultured human hair follicles and augment the hair-inductive capacity of cultured dermal papilla spheres. Exp. Dermatol..

[96] Hu S (2020). Dermal exosomes containing miR-218-5p promote hair regeneration by regulating beta-catenin signaling. Sci. Adv..

[97] Wang J (2018). Bottom-up nanoencapsulation from single cells to tunable and scalable cellular spheroids for hair follicle regeneration. Adv. Healthc. Mater.

[98] Zhang X (2019). Use of extracellular matrix hydrogel from human placenta to restore hair-inductive potential of dermal papilla cells. Regen. Med..

[99] Vahav I (2020). Reconstructed human skin shows epidermal invagination towards integrated neopapillae indicating early hair follicle formation in vitro. J. Tissue Eng. Regen. Med..

[100] Zhang K (2020). Cellular nanofiber structure with secretory activity-promoting characteristics for multicellular spheroid formation and hair follicle regeneration. ACS Appl. Mater. Interfaces.

[101] Abaci HE (2018). Tissue engineering of human hair follicles using a biomimetic developmental approach. Nat. Commun..

[102] Wang X (2016). Self-assembling peptide hydrogel scaffolds support stem cell-based hair follicle regeneration. Nanomedicine.

[103] Wang X (2016). Hair follicle and sebaceous gland de novo regeneration with cultured epidermal stem cells and skin-derived precursors. Stem Cells Transl. Med..

[104] Ellis-Behnke RG (2006). Nano neuro knitting: peptide nanofiber scaffold for brain repair and axon regeneration with functional return of vision. Proc. Natl Acad. Sci. USA.

[105] Meng H (2009). The effect of a self-assembling peptide nanofiber scaffold (peptide) when used as a wound dressing for the treatment of deep second degree burns in rats. J. Biomed. Mater. Res. B.

[106] Kumada Y, Zhang S (2010). Significant type I and type III collagen production from human periodontal ligament fibroblasts in 3D peptide scaffolds without extra growth factors. PLoS ONE.

[107] Carrejo NC (2018). Multidomain peptide hydrogel accelerates healing of full-thickness wounds in diabetic mice. ACS Biomater. Sci. Eng..

[108] Zhang Y (2019). Multifunctional Zn doped hollow mesoporous silica/polycaprolactone electrospun membranes with enhanced hair follicle regeneration and antibacterial activity for wound healing. Nanoscale.

[109] Bader A (2012). Skin regeneration with conical and hair follicle structure of deep second-degree scalding injuries via combined expression of the EPO receptor and beta common receptor by local subcutaneous injection of nanosized rhEPO. Int. J. Nanomed..

[110] Giri P (2015). Skin regeneration in deep second-degree scald injuries either by infusion pumping or topical application of recombinant human erythropoietin gel. Drug Des. Dev. Ther..

[111] Zhang Z (2020). Design of a multifunctional biomaterial inspired by ancient chinese medicine for hair regeneration in burned skin. ACS Appl. Mater. Interfaces.

[112] Clevers H (2016). Modeling development and disease with organoids. Cell.

[113] Gupta AC (2018). Establishment of an in vitro organoid model of dermal papilla of human hair follicle. J. Cell. Physiol..

[114] Lee J (2018). Hair follicle development in mouse pluripotent stem cell-derived skin organoids. Cell Rep..

[115] Su Y (2019). Pre-aggregation of scalp progenitor dermal and epidermal stem cells activates the WNT pathway and promotes hair follicle formation in in vitro and in vivo systems. Stem Cell Res. Ther..

[116] Takagi R (2016). Bioengineering a 3D integumentary organ system from iPS cells using an in vivo transplantation model. Sci. Adv..

[117] Stenn KS, Paus R (2001). Controls of hair follicle cycling. Physiol. Rev..

[118] Hebert JM, Rosenquist T, Gotz J, Martin GR (1994). FGF5 as a regulator of the hair growth cycle: evidence from targeted and spontaneous mutations. Cell.

[119] Palmer HG, Martinez D, Carmeliet G, Watt FM (2008). The vitamin D receptor is required for mouse hair cycle progression but not for maintenance of the epidermal stem cell compartment. J. Invest. Dermatol..

[120] Watt FM, Estrach S, Ambler CA (2008). Epidermal Notch signalling: differentiation, cancer and adhesion. Curr. Opin. Cell Biol..

[121] Suen WJ, Li ST, Yang LT (2020). Hes1 regulates anagen initiation and hair follicle regeneration through modulation of hedgehog signaling. Stem Cells.

[122] Legrand JMD (2016). STAT5 activation in the dermal papilla is important for hair follicle growth phase induction. J. Invest. Dermatol..

[123] Watabe R (2014). Leptin controls hair follicle cycling. Exp. Dermatol..

[124] Sumikawa Y, Inui S, Nakajima T, Itami S (2014). Hair cycle control by leptin as a new anagen inducer. Exp. Dermatol..

[125] Nakajima T (2013). Roles of MED1 in quiescence of hair follicle stem cells and maintenance of normal hair cycling. J. Invest. Dermatol..

[126] Zhu K, Xu C, Liu M, Zhang J (2017). Hairless controls hair fate decision via Wnt/beta-catenin signaling. Biochem. Biophys. Res. Commun..

[127] Saini V (2017). Absence of vitamin D receptor (VDR)-mediated PPARgamma suppression causes alopecia in VDR-null mice. FASEB J..

[128] Mercati F (2019). Epithelial expression of the hormone leptin by bovine skin. Eur. J. Histochem..

[129] Tong X, Coulombe PA (2006). Keratin 17 modulates hair follicle cycling in a TNFalpha-dependent fashion. Genes Dev..

[130] Zhou L (2018). Decorin promotes proliferation and migration of ORS keratinocytes and maintains hair anagen in mice. Exp. Dermatol..

[131] Qiu W (2016). Hoxc13 is a crucial regulator of murine hair cycle. Cell Tissue Res..

[132] Jing J (2014). Expression of decorin throughout the murine hair follicle cycle: hair cycle dependence and anagen phase prolongation. Exp. Dermatol..

[133] Wu XJ (2012). Expression and location of phospho-Artemis (Serine516) in hair follicles during induced growth of mouse hair. Arch. Dermatol. Res..

[134] Samuelov L (2012). P-cadherin regulates human hair growth and cycling via canonical Wnt signaling and transforming growth factor-beta2. J. Invest. Dermatol..

[135] Semenova E (2008). Overexpression of mIGF-1 in keratinocytes improves wound healing and accelerates hair follicle formation and cycling in mice. Am. J. Pathol..

[136] Bikle DD (2006). Development and progression of alopecia in the vitamin D receptor null mouse. J. Cell. Physiol..

[137] Panteleyev AA (1998). Towards defining the pathogenesis of the hairless phenotype. J. Invest. Dermatol..

[138] Potter GB (2001). The hairless gene mutated in congenital hair loss disorders encodes a novel nuclear receptor corepressor. Genes Dev..

[139] Al-Nuaimi Y (2014). A meeting of two chronobiological systems: circadian proteins Period1 and BMAL1 modulate the human hair cycle clock. J. Invest. Dermatol..

[140] Castela M (2017). Igf1r signalling acts on the anagen-to-catagen transition in the hair cycle. Exp. Dermatol..

[141] Bichsel KJ (2016). The epidermal growth factor receptor decreases Stathmin 1 and triggers catagen entry in the mouse. Exp. Dermatol..

[142] Coulson-Thomas VJ, Gesteira TF, Esko J, Kao W (2014). Heparan sulfate regulates hair follicle and sebaceous gland morphogenesis and homeostasis. J. Biol. Chem..

[143] Kim BK (2014). Increased expression of Dkk1 by HR is associated with alteration of hair cycle in hairpoor mice. J. Dermatol. Sci..

[144] Kwack MH, Kim MK, Kim JC, Sung YK (2012). Dickkopf 1 promotes regression of hair follicles. J. Invest. Dermatol..

[145] Kandyba E, Kobielak K (2014). Wnt7b is an important intrinsic regulator of hair follicle stem cell homeostasis and hair follicle cycling. Stem Cells.

[146] Bai X (2015). Roles of GasderminA3 in catagen–telogen transition during hair cycling. J. Invest. Dermatol..

[147] Plikus MV (2008). Cyclic dermal BMP signalling regulates stem cell activation during hair regeneration. Nature.

[148] Wu P (2019). The balance of Bmp6 and Wnt10b regulates the telogen-anagen transition of hair follicles. Cell Commun. Signal..

[149] Calvo-Sanchez MI (2019). A role for the Tgf-beta/Bmp co-receptor Endoglin in the molecular oscillator that regulates the hair follicle cycle. J. Mol. Cell. Biol..

[150] Krieger K (2018). NF-kappaB participates in mouse hair cycle control and plays distinct roles in the various pelage hair follicle types. J. Invest. Dermatol..

